# Lymph node ratio as valuable predictor in pancreatic cancer treated with R0 resection and adjuvant treatment

**DOI:** 10.1186/s12885-019-6193-0

**Published:** 2019-10-15

**Authors:** Min Su You, Sang Hyub Lee, Young Hoon Choi, Bang-sup Shin, Woo Hyun Paik, Ji Kon Ryu, Yong-Tae Kim, Dong Kee Jang, Jun Kyu Lee, Wooil Kwon, Jin-Young Jang, Sun-Whe Kim

**Affiliations:** 1 Department of Internal Medicine and Liver Research Institute, Seoul National University College of Medicine, Seoul National University Hospital, 101, Daehak-ro, Jongno-gu, 03080 Seoul, Republic of Korea; 2 Department of Internal Medicine, Dongguk University Ilsan Hospital, Gyeonggi-do, Goyang-si, South Korea; 3 Department of Surgery, Seoul National University College of Medicine, Seoul National University Hospital, Seoul, South Korea

**Keywords:** Pancreatic cancer, Adjuvant therapy, Lymph nodes, Metastasis, Prognosis

## Abstract

**Background:**

Lymph-node (LN) metastasis is an important prognostic factor in resected pancreatic cancer. In this study, the prognostic value of American Joint Committee on Cancer (AJCC) 8th edition N stage, lymph-node ratio (LNR), and log odds of positive lymph nodes (LODDS) in resected pancreatic cancer was investigated.

**Methods:**

Between January 2005 and December 2017, there were 351 patients with pancreatic cancer treated with R0 resection and adjuvant therapy at Seoul National University Hospital. Relationships between the three LN parameters and overall survival (OS) and recurrence-free survival (RFS) were evaluated using a log-rank test and Cox proportional hazard regression model. Each multivariate-adjusted LN parameter was internally validated by bootstrap-corrected Harrell’s C-index.

**Results:**

The mean duration from surgery to adjuvant therapy was 47.6 ± 17.4 days. In total, the median OS and RFS was 31.7 (95% CI, 27.2-37.2) and 15.4 (95% CI, 13.5-17.7) months. The three LN classification systems were significantly correlated with OS and RFS in log-rank tests and multivariate-adjusted models (all *p* < 0.05). When internally validated, LNR showed the highest discrimination ability in predicting OS and RFS (each *C*–index = 0.65). LNR also showed the highest C-index in subgroup analysis, classified by adjuvant therapy modality. LNR and the AJCC 8th edition LN classification system were significantly associated with loco-regional recurrence (*p* = 0.026 and *p* = 0.027, respectively).

**Conclusions:**

LNR, which showed the best prognostic performance and significant relationship with loco-regional recurrence, can help further stratify the patients and establish an active treatment plan.

## Background

Pancreatic cancer is currently the third greatest cause of cancer-related death and has a five-year survival rate of 8% [1]. Surgical treatment is the only curative method, but less than 20% of the patients can be operated on when diagnosed [2]. Furthermore, the recurrence rate reaches 60-80% even with surgery [3]. The lymph-node (LN) status is an important predictor of recurrence and survival in surgically treated pancreatic cancer, and LN status evaluation is generally based on the American Joint Committee on Cancer (AJCC) classification system. In the AJCC 7th edition, the staging system defined all regional LN metastases as N1. The 8th edition was revised to further evaluate the LN grade based on the number of metastatic nodes [4].

Previous studies have consistently reported that LN metastasis is closely related to prognosis in pancreatic cancer treated with surgery [5–7]. In addition to the AJCC staging system, lymph-node ratio (LNR) and log odds of positive lymph nodes (LODDS) are also well-known parameters of LN metastasis. Recently, Vicente et al. [6] evaluated the prognostic role of various LN classification systems, including the N stage of the AJCC 7th and 8th editions, LNR, and LODDS in surgically resected pancreatic-cancer patients. Previous studies have shown that the prognostic value of each LN classification system is affected by the resection margin [6, 7]. However, there are limited data about the diverse LN staging systems in terms of pancreatic cancer treated with R0 resection.

Postoperative adjuvant therapy significantly increases survival and is indispensable in patients with pancreatic cancer after surgery [8]. In real clinical practice, there is considerable diversity in the methods of adjuvant therapy, since previous studies have reported the efficacy of various adjuvant chemotherapy and chemoradiation therapy regimens [8]. The most recent clinical trial on adjuvant chemotherapy for pancreatic cancer, the PRODIGE 24/CCTG PA.6 trial, reported a median survival of 54.4 months and a three-year survival rate of 63.4% in patients receiving a modified regimen of oxaliplatin, irinotecan, fluorouracil, and leucovorin (FOLFIRINOX) after curative surgery [9]. Although postoperative adjuvant treatment has become the standard therapy, most of the previous studies have investigated the prognostic value of LN metastasis in surgically treated pancreatic-cancer patients regardless of the adjuvant treatment [5–7].

In general, the AJCC staging system is widely used for evaluation of LN metastasis. Since there are usually fewer than four LN metastases among most of the patients treated with R0 resection [10, 11], further stratification by various LN classification systems can be helpful. There is still a lack of studies evaluating the several LN classification systems in resected pancreatic cancer. Therefore, this study aims to evaluate the prognostic performance of the various LN staging systems, including the AJCC 8th edition N stage, LNR, and LODDS in patients with pancreatic cancer treated with R0 resection and subsequent adjuvant treatment.

## Methods

### Study population

**Fig. 1:**
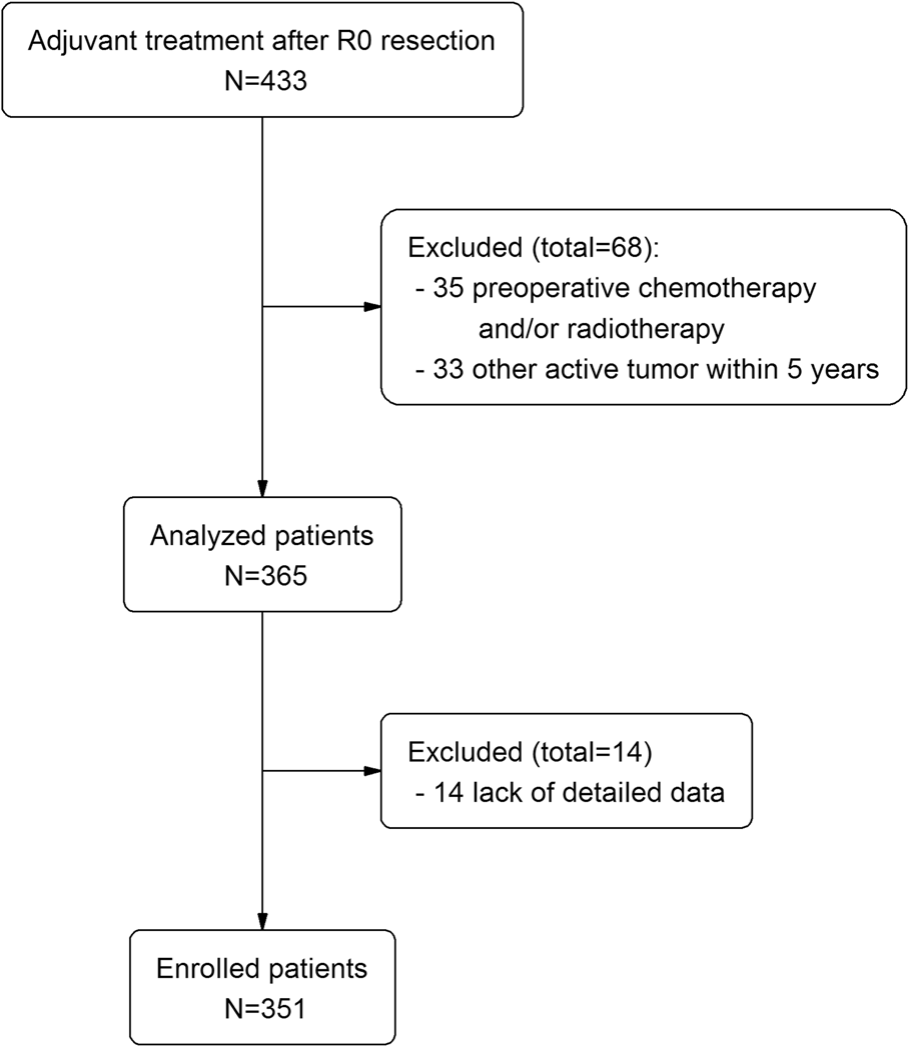
Flowchart of patient enrollment. PDAC, pancreatic ductal adenocarcinoma; LN, lymph node

From January 2005 to December 2017, 433 patients with pancreatic cancer received adjuvant treatment after R0 resection at Seoul National University Hospital. Range of surgery included pancreaticoduodenectomy, distal pancreatectomy, central pancreatectomy, and total/subtotal pancreatectomy. All patients were confirmed to have pancreatic ductal adenocarcinoma by pathologic examination. After exclusion of 35 patients who had undergone preoperative chemotherapy and/or radiotherapy, and 33 patients with a history of other active tumors within five years, medical records of the remaining 365 patients were reviewed. Excluding another 14 patients who lacked details of pathology and laboratory findings, we enrolled a total of 351 patients (Fig. 1). This study was conducted under the approval of the Institutional Review Board of Seoul National University Hospital, Seoul, Korea (1802-113-924).

### Data collection

Patient demographics, body mass index, Eastern Cooperative Oncology Group (ECOG) performance status, Charlson comorbidity index, and laboratory findings before surgery were investigated. A contrast-enhanced computed tomography (CT) scan was used to evaluate the location, size, and extent of the tumor. All patients underwent adjuvant treatment within four months after the surgery. Type and treatment protocols of adjuvant treatment were chosen after discussion with multidisciplinary clinicians and the patient.

The onset of follow-up was set to the date of surgery. One month after the operation and three months after the initial adjuvant treatment, patients were assessed for recurrence using a contrast-enhanced CT scan. Patients were followed up at three- to six-month intervals in the first two years and then at six- to twelve-month intervals from the third year if there was no evidence of tumor recurrence. In patients with confirmed recurrence after surgery, ^18^F-fluorodeoxyglucose-positron emission tomography was performed to evaluate distant recurrence in addition to the contrast-enhanced CT scan.

Based on pathologic findings, the total number of dissected lymph nodes and the number of metastatic lymph nodes, tumor location, diameter, histologic differentiation, AJCC 8th edition of TNM stage, resection margin status, lymphovascular invasion, and perineural invasion were evaluated. The LNR was calculated as the number of positive LNs divided by the number of LNs examined and was categorized as 0, 0.01–0.20, 0.21–0.40, and > 0.40 on the basis of previously proposed cut-off values [12]. The LODDS was estimated by log [(number of positive LNs + 0.5)/(total number of LNs examined – number of positive LNs + 0.5)] and divided into four groups according to quartiles.

### Outcome measures

Overall survival (OS) was defined from the date of surgery to the date of death from any cause. Data on deaths were collected from the database of the Korean Ministry of the Interior and Safety. Recurrence-free survival (RFS) was defined as time from the curative surgery to the tumor recurrence. Recurrence was confirmed radiologically and/or histologically. Patients who were alive at the last date of follow-up without tumor recurrence and who died without any evidence of tumor recurrence were censored for RFS.

### Statistical analysis

Data are presented as prevalence (%) or median with range. Student’s t test was performed to compare continuous variables, and the chi-square test was used to compare categorical data. Survival was analyzed by the Kaplan–Meier method and expressed with median and 95% confidence interval (CI). Survival between groups was compared using a log-rank test and a Cox proportional hazard regression model.

All three LN staging systems were evaluated using the categorical cut-off values, and associations of the LN parameters with OS and RFS were analyzed. The LN parameters were adjusted by age, sex, and several covariates that showed *p* ≤ 0.2 in univariate Cox proportional hazard regression analysis. With these parameters, a best-fitted multivariate model for each LN classification system was selected using Akaike information criterion (AIC)-based backward selection. The three prognostic models were internally validated by bootstrap-corrected Harrell’s *C*–index 0.5 (no discrimination) to 1 (perfect discrimination); bootstrapping with 200 resamples was performed [13]. Interactions were evaluated by including cross-product interaction terms in the multivariate regression models. All statistics were evaluated using R version 3.5.0 for Windows (Institute for Statistics and Mathematics, Vienna, Austria; http://www.R-project.org).

## Results

### Patient characteristics

Baseline clinical and histologic characteristics are shown in Table 1. The mean age of the patients at surgery was 63.3 ± 9.3 years. Tumors were mainly located in the head/uncinate area (64.1%), and pancreaticoduodenectomy was the most commonly implemented procedure (66.1%). The number of examined LNs averaged 18.4 ± 11.7 in the entire cohort, 20.9 ± 12.5 in patients with total/subtotal pancreatectomy, 20.0 ± 12.0 in patients with pancreaticoduodenectomy, and 14.7 ± 10.0 in patients with distal/central pancreatectomy. There was a significant difference in the number of examined LNs between the surgery types. (*p* = 0.001). All patients were confirmed with microscopic complete resection, and the median follow-up duration after surgery was 31.1 ± 27.3 months.

**Table 1:** Clinicopathological characteristics of patients who underwent R0 resection and adjuvant therapy for pancreatic cancer

Variables	*N* = 351
Age	63.3 ± 9.3
Sex	
- Female	148 (42.2%)
- Male	203 (57.8%)
Body mass index, kg/m^2^	22.9 ± 2.7
ECOG	
- 0	289 (82.3%)
- 1	62 (17.7%)
Charlson’s comorbidity index	4.6 ± 1.3
Tumor location	
- Head/uncinate	225 (64.1%)
- Body	62 (17.7%)
- Tail	52 (14.8%)
- Overlapping	12 (3.4%)
Surgery type	
- Pancreaticoduodenectomy	232 (66.1%)
- Distal pancreatectomy	107 (30.5%)
- Total	7 (2.0%)
- Subtotal	4 (1.1%)
- Central pancreatectomy	1 (0.3%)
Histologic grade	
- Well differentiated	28 (8.0%)
- Moderately differentiated	289 (82.3%)
- Poorly differentiated	31 (8.8%)
- Undifferentiated	3 (0.9%)
pT stage (AJCC 8th)	
- 1a / 1b / 1c	2 (0.6%) / 4 (1.1%) / 61 (17.4%)
- 2	235 (67.0%)
- 3	45 (12.8%)
- 4	4 (1.1%)
Number of examined LNs	18.4 ± 11.7
Number of involved LNs	1.8 ± 2.6
pN stage (AJCC 8th)	
- 0	150 (42.7%)
- 1	135 (38.5%)
- 2	66 (18.8%)
LNR	0.1 ± 0.2
LODDS	−1.0 ± 0.5
Lymphovascular invasion	158 (45.0%)
Perineural invasion	293 (83.5%)
Preoperative laboratory findings	
- WBC, 10^3^/μL	6.2 ± 1.9
- Hemoglobin, g/dL	12.9 ± 1.5
- Platelet, 10^3^/μL	248.0 ± 79.5
- Albumin, g/dL	4.2 ± 3.4
- Total bilirubin, mg/dL	2.2 ± 3.5
- AST, IU/L	43.1 ± 49.0
- ALT, IU/L	65.7 ± 92.6
- ALP, IU/L	157.0 ± 162.7
- CA 19-9, U/mL	642.3 ± 1563.6
- CEA, ng/mL	21.5 ± 300.0

*ECOG* Eastern Cooperative Oncology Group, *AJCC* American Joint Committee on Cancer, *LNR* lymph-node ratio, *LODDS* log odds of positive lymph nodes, *WBC* white blood cell, *AST* aspartate Aminotransferase, *ALT* alanine aminotransferase, *ALP* alkaline phosphatase, *CA 19-9* carbohydrate antigen 19-9, *CEA* carcinoembryonic antigen

### Adjuvant treatment regimens

The mean duration from surgery to adjuvant therapy was 47.6 ± 17.4 days. Details of adjuvant treatment are summarized in Additional file 1: Table S1. A total of 263 patients (74.9%) underwent adjuvant concurrent chemoradiation therapy (CCRT). CCRT with induction and/or maintenance chemotherapy was conducted for 199 (56.7%) patients, whereas 64 (18.2%) patients underwent CCRT without additive chemotherapy. The most common chemotherapeutic agent of CCRT was 5-fluorouracil (163/263, 62.0%), followed by gemcitabine (90/263, 34.2%) and capecitabine (10/263, 3.8%). Induction chemotherapy was performed in 60 (17.1%) patients, and all of them received gemcitabine-based chemotherapy. Maintenance chemotherapy was performed for 186 (53.0%) patients, gemcitabine-based chemotherapy for 119 (64.0%) patients, and fluorouracil-based chemotherapy for 67 (36.0%) patients.

Adjuvant treatment with chemotherapy alone was performed for 88 (25.1%) patients; the chemotherapy regimens included gemcitabine-based chemotherapy (63/88, 71.6%), Fluorouracil-based chemotherapy (24/88, 27.3%), and oral tegafur (1/88, 1.1%). Second-line adjuvant therapy was performed for 82 (23.4%) patients; there was gemcitabine-based chemotherapy for 51 (62.2%) patients, fluorouracil-based chemotherapy for 30 (36.6%) patients, and oral tegafur for 1 (1.2%) patient. The median OS of the patients treated with CCRT and chemotherapy alone was 32.1 (95% CI, 27.0-39.2) and 30.5 (95% CI, 26.9-NE) months, respectively. There was no statistical difference between the two groups (*p* = 0.960). During follow-up, recurrence was confirmed in 222 (63.2%) patients. Palliative chemotherapy was most commonly performed (164/222, 73.9%) after recurrence, followed by best supportive care (21/222, 9.5%), radiotherapy (9/222, 4.1%), and surgical therapy (5/222, 2.3%). A few (23/222, 10.4%) patients were lost to follow-up after recurrence.

### Prognostic performance of LN staging systems

**Fig. 2:**
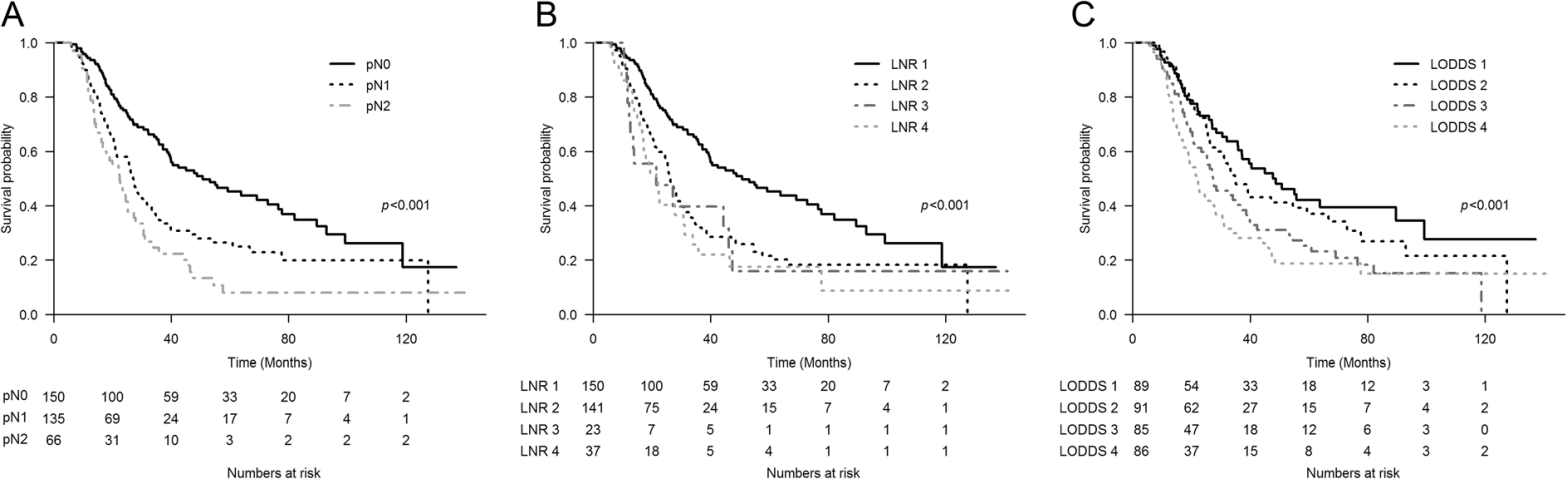
Overall survival graph by Kaplan–Meier survival method, classified by (**a**) AJCC 8th edition N stage, (**b**) LNR (LNR 1, LNR = 0; LNR 2, LNR > 0 to ≤0.2; LNR 3, LNR > 0.2 to ≤0.4; LNR 4, LNR > 0.4), and (**c**) LODDS (LODDS 1, quantile 1; LODDS 2, quantile 2; LODDS 3, quantile 3; LODDS 4, quantile 4). AJCC, American Joint Committee on Cancer; LNR, Lymph-node ratio; LODDS, log odds of metastatic lymph nodes

The median OS and RFS was 31.7 (95% CI, 27.2-37.2) and 15.4 (95% CI, 13.5-17.7) months, respectively. Figure 2 shows the relationships between OS and AJCC N stage, LNR, and LODDS. All three LN staging systems were significantly correlated with OS (*p* < 0.001). In the univariate Cox proportional hazard regression model predicting OS, five covariates showed *p* ≤ 0.2; these were histologic grade, lymphovascular invasion, perineural invasion, pathologic T stage, and preoperative CA19-9 ≥ 100 U/mL. When OS and RFS were evaluated by a multivariate-adjusted LN model that included the above covariates, all three LN classification systems were significantly correlated with OS and RFS (Table 2). As the LN grade increased, the predicted hazard ratio (HR) of OS and RFS increased proportionally in the three LN staging systems. Additionally, each multivariate-adjusted LN prognostic model was internally validated (Table 2). LNR showed the highest discrimination ability in predicting OS and RFS.

**Table 2:** Multivariate-adjusted Cox proportional hazard regression models predicting OS and RFS classified by AJCC 8th edition N stage, LNR, and LODDS

	Number of patients	OS					RFS				
		Median OS, months	HR (95% CI) ^a^	*p*	AIC	Bootstrap-corrected *C* index	Median RFS, months	HR (95% CI) ^a^	*p*	AIC	Bootstrap-corrected *C* index
pN (AJCC 8th)											
0	150	50.8	1	< 0.001	2003.0	0.57	23.5	1	< 0.001	2274.3	0.57
1	135	27.0	1.58 (1.14-2.19)				15.0	1.34 (0.98-1.83)			
2	66	22.5	2.00 (1.36-2.95)				10.1	2.01 (1.38-2.92)			
LNR											
0	150	50.8	1	< 0.001	2005.9	0.65	23.5	1	0.001	2278.4	0.63
> 0 to ≤0.2	141	26.3	1.62 (1.17-2.23)				13.5	1.39 (1.02-1.88)			
> 0.2 to ≤0.4	23	21.5	1.66 (0.90-3.01)				11.0	1.80 (1.02-3.18)			
> 0.4	37	21.4	2.05 (1.31-3.20)				9.2	1.90 (1.23-2.96)			
LODDS											
Quantile 1	89	48.8	1	0.002	2008.8	0.64	20.6	1	0.009	2277.5	0.63
Quantile 2	91	34.8	1.26 (0.84-1.91)				17.3	1.17 (0.79-1.72)			
Quantile 3	85	27.4	1.46 (0.97-2.20)				15.4	1.24 (0.84-1.84)			
Quantile 4	86	22.0	1.90 (1.25-2.87)				10.1	1.78 (1.21-2.62)			

*OS* overall survival, *RFS* recurrence-free survival, *AIC* akaike information criterion, *AJCC* American Joint Committee on Cancer, *LNR* lymph-node ratio, *LODDS* log odds of positive lymph nodes, *CA 19-9* carbohydrate antigen 19-9

^a^ HR adjusted with the histologic grade, lymphovascular invasion, perineural invasion, pathologic T stage, and preoperative CA19-9 ≥ 100 U/mL

Of the 222 patients with recurrences, a tumor was identified in a local region in 55 (24.8%) patients. The association of different LN staging systems with loco-regional recurrence was further analyzed by a multivariate-adjusted model using HR and 95% CI (Table 3). LNR and AJCC N stage were significantly associated with loco-regional recurrence (*p* = 0.026 and *p* = 0.027, respectively). There was no LN staging classification system that was significantly correlated with distant recurrence.

**Table 3:** Predictive hazard ratio of local recurrence by three multivariate-adjusted lymph-node classification models

	Local recurrence (*N* = 55)		
	Number of patients	HR (95% CI) ^a^	*p*
pN (AJCC 8th)			0.027
0	16	1.00	
1	22	1.44 (0.90-2.32)	
2	17	1.85 (1.05-3.24)	
LNR			0.026
0	16	1.00	
> 0 to ≤0.2	3	1.43 (0.86-2.35)	
> 0.2 to ≤0.4	15	2.26 (0.94-5.44)	
> 0.4	21	1.96 (0.99-3.90)	
LODDS			0.138
Quantile 1	10	1.00	
Quantile 2	14	1.08 (0.60-1.95)	
Quantile 3	13	0.61 (0.31-1.18)	
Quantile 4	18	1.54 (0.86-2.76)	

*AJCC* American Joint Committee on Cancer, *LNR* lymph-node ratio, *LODDS* log odds of positive lymph nodes, *CA 19-9* carbohydrate antigen 19-9

^a^ HR adjusted with the histologic grade, lymphovascular invasion, perineural invasion, pathologic T stage, and preoperative CA19-9 ≥ 100 U/mL

There were 169 patients in low-LNR group (LNR ≤ 0.2) and 182 patient in high-LNR group (LNR > 0.2). In subgroup analysis based on number of 12 examined LNs in low-LNR group, there was no significant difference in OS (59.3 [95% CI, 39.2-93.0] months vs 40.5 [95% CI, 32.1-63.8] months; *p* = 0.337), RFS (29.1 [95% CI, 16.8-NA] months vs 18.8 [95% CI, 14.2-37.3] months; *p* = 0.333), and recurrence rate (54.2% vs 58.8%; *p* = 0.660) between the two groups. Likewise, subgroup analysis in high-LNR group showed no significant difference in OS (21.5 [95% CI, 18.0-39.8] months vs 25.7 [95% CI, 22.0-30.5] (95% CI, 10.3-15.3) months; *p* = 0.897), RFS (15.0 [95% CI, 10.8-25.9] months vs 12.1 [95% CI, 10.3-15.3] months; *p* = 0.224), and recurrence rate (61.5% vs 72.3%, *p* = 0.213).

When subgroup analysis of patients who underwent CCRT was performed, the predictive performance of LNR was highest (*C*–index = 0.63), followed by LODDS (*C*–index = 0.62) and AJCC N stage (*C*–index = 0.57). Likewise, in subgroup analysis in patients who underwent only chemotherapy postoperatively, LNR showed the best prognostic performance (*C*–index = 0.67), followed by LODDS (*C*–index = 0.65) and AJCC N stage (*C*–index = 0.58).

## Discussion

LN status is an important factor for predicting the prognosis of pancreatic cancer after surgery [5, 6, 14, 15]. LN metastasis can be evaluated in several ways based on the presence, number, and ratio of metastatic lymph nodes [6, 7]. In this study, the associations of the AJCC 8th edition N stage, LODDS, and LNR with prognosis in pancreatic cancer treated with adjuvant treatment after R0 resection were analyzed. All three parameters were significantly correlated with OS and RFS. LNR and AJCC N stage were statistically related to the loco-regional recurrence. LNR demonstrated the highest discrimination ability in predicting OS and RFS in both internal validation and subgroup analysis.

In this study, LN metastases was fewer than four in ~ 80% of the patients, and ~ 40% of the patients had no LN metastasis. The number of examined LNs may differ even in patients with the same number of LN metastases at the time of surgery; the actual prognosis may differ between the patients, but the same grade is given in the AJCC 8th edition N stage. MLODDS and LNR intrinsically evaluate not only the number of LN metastases but also the number of examined LNs. Both of them are advantageous for evaluating the adequacy of LN dissection and are less vulnerable to stage migration [16, 17]. In addition, since LODDS uses log odds, even patients without LN metastasis can be further subdivided [17]. Using LNR and LODDS will help overcome the shortcomings of the AJCC N stage and further stratify the PDAC treated with R0 resection and adjuvant treatment.

Currently, Vincente et al. [6] assessed the association of several LN classification systems with local recurrence based on logistic regression analysis and found no association between them. On the other hand, LNR and AJCC N stage were significantly related to the loco-regional recurrence in this study. These two LN classification systems are expected to help further classify patients and establish an active treatment plan such as radiotherapy in patients at high risk for loco-regional recurrence. Application of stereotactic ablative radiotherapy, which is known to be more potent than is conventional RT, can also be considered for loco-regional control [18, 19].

In a previous study using the Surveillance, Epidemiology, and End Results (SEER) database, the median number of examined LNs in patients with resected pancreatic cancer was 7 (range, 0-90), and at least 12 resected lymph nodes were required to adequately evaluate LN metastasis [14]. It is also known that the number of examined LNs in pancreatic cancer can be different according to the type of surgery [4, 6]. In this study, the number of dissected LNs was more than 12 regardless of the surgery type. It is suggested that if enough LN dissections are done, different LN staging systems can be applied universally regardless of the type of surgery.

A variety of therapeutic regimens have been actively studied as adjuvant treatment for resected pancreatic cancer, and there is no standard treatment protocol yet [9, 20–22]. The recent study demonstrated that the modified-FOLFIRINOX regimen showed significantly longer survival than did gemcitabine among patients with resected pancreatic cancer [9]. In addition, a clinical trial regarding nab-paclitaxel and gemticabine in resected pancreatic cancer is ongoing (NCT01964430) [23]. Because the treatment duration, dosage, effect, and toxicity all depend on the type of treatment regimens, multidisciplinary discussion is essential in deciding on the appropriate treatment protocol for each patient. In this study, most of the patients were treated with CCRT after R0 resection, and there was no significant difference in overall survival between the CCRT and chemotherapy groups. When subgroup analysis was performed according to CCRT and chemotherapy, each LN system showed a prognostic performance comparable to that of the entire cohort. Regardless of the type of adjuvant therapy, LN metastasis status can help further stratify patients and find aggressive treatment strategies, such as the latest chemotherapy and clinical trials.

This study has several limitations. First, it is a single-center, retrospective study with a relatively small number of patients. The number of examined LNs is known to affect the prognosis, but this study did not show statistically significant results. In this study, margin-positive patients who may have poor prognosis were excluded in the first place, resulting in selection bias. In addition, LODDS was associated with loco-regional recurrence, but not statistically significant. This may stem from the small sample size and caution should be exercised in interpreting the results. Second, it did not include an external validation cohort. However, internal validation using a bootstrapping-adjusted *C*–index was applied to overcome the lack of an external validation set. A well-designed prospective validation study with a large number of patients is required to adequately assess the prognostic value of the LN prognostic models. Third, the diversity of CCRT and chemotherapy regimens may serve as a confounding factor. Fourth, for the cut-off value of LNR, this study defined LNR on the basis of the most recent study [6]. However, the optimal cut-off value has not yet been established [24–27]. Well-designed, large prospective studies are needed to find the optimal LNR cut-off value predicting prognosis in resected pancreatic cancer.

## Conclusions

Various LN classification systems, including the AJCC 8th edition N stage, LNR, and LODDS demonstrated significant prognostic performance in R0 resected pancreatic cancer. LNR showed not only the best prognostic performance but also a significant relationship with loco-regional recurrence. Further stratification according to LNR may help establish an active treatment plan and predict loco-regional recurrence in patients with pancreatic cancer treated with R0 resection and adjuvant treatment.

## Supplementary information

**Additional file 1: Table S1.** Detailed data regarding adjuvant treatment.

## Data Availability

The datasets used and/or analysed during the current study are available from the corresponding author on reasonable request.

## Abbreviations

AIC: Akaike information criterion
AJCC: American Joint Committee on Cancer
CCRT: Concurrent chemoradiation therapy
CI: Confidence interval
CT: Computed tomography
ECOG: Eastern Cooperative Oncology Group
LN: Lymph-node
LNR: Lymph-node ratio
LODDS: Log odds of positive lymph nodes
OS: Overall survival
RFS: Recurrence-free survival
