# Supplementary material for: Lymph node ratio as valuable predictor in pancreatic cancer treated with R0 resection and adjuvant treatment
Source: BMC Cancer. 2019 Oct 15;19:952. doi: 10.1186/s12885-019-6193-0 (PMC6794802; doi:10.1186/s12885-019-6193-0)
Supplement: Supplementary file 1 — Additional file 1: Table S1. Detailed data regarding adjuvant treatment. [file 12885_2019_6193_MOESM1_ESM.docx]

Additional file 1: Table S1. Detailed data regarding adjuvant treatment

| Treatment data | N=351 |
| --- | --- |
| Type of adjuvant therapy |  |
| - CCRT only | 66 (18.8%) |
| - CCRT and chemotherapy | 197 (56.1%) |
| - Induction chemotherapy | 60 (17.1%) |
| - Gemcitabine-based | 60 (17.1%) |
| - Maintenance chemotherapy | 183 (52.1%) |
| - Gemcitabine-based | 116 (33.0%) |
| - 5-FU-based | 67 (19.1%) |
| - Chemotherapy only | 88 (25.1%) |
| - Gemcitabine-based | 63 (71.6%) |
| - 5-FU-based | 24 (6.8%) |
| - Oral tegafur | 1 (0.3%) |
| Chemotherapeutic agent of CCRT |  |
| - 5-FU | 163 (46.4%) |
| - Gemcitabine | 90 (25.6%) |
| - Capecitabine | 10 (2.8%) |
| Second-line adjuvant chemotherapy | 82 (23.4%) |
| - Gemcitabine-based | 51 (14.5%) |
| - 5-FU-based | 30 (8.5%) |
| - Oral tegafur | 1 (0.3%) |
| Follow-up duration, months | 31.1 ± 27.3 |

CCRT, concurrent chemoradiation therapy; 5-FU, 5-fluorouracil
